# Biodegradable Protein Films Derived from Pea-Processing By-Products: Matrix Compositions, Properties, and Potential in Active Food Packaging

**DOI:** 10.3390/molecules31173061

**Published:** 2026-08-31

**Authors:** Kübra Altuntaş, Ayşe Saygün

**Affiliations:** Food Engineering Department, Chemical and Metallurgical Engineering Faculty, Istanbul Technical University, Istanbul 34469, Türkiye; kubraaltuntas@itu.edu.tr

**Keywords:** pea protein, biodegradable films, matrix, active packaging, antimicrobial, antioxidant

## Abstract

The extensive use of petroleum-based packaging materials has generated significant environmental concerns because of their poor biodegradability and their contribution to plastic pollution. Growing attention has therefore been directed toward sustainable packaging systems derived from renewable resources. Among these alternatives, proteins recovered from pea-processing by-products have emerged as promising film-forming materials because of their biodegradability, renewability, and compatibility with circular-bioeconomy principles. This review critically synthesises, rather than merely catalogues, the development of pea protein-based films produced with different biopolymer matrices, with emphasis on fabrication methods, film-formation mechanisms, physicochemical and mechanical properties, barrier performance, and antimicrobial/antioxidant functionality. Where the literature reports conflicting or concentration-dependent results—for example, the divergent effect of essential oil type on tensile strength, or the existence of optimal polyphenol-loading thresholds beyond which mechanical and barrier performance deteriorate—mechanistic explanations grounded in protein additive interactions, cross-link density, and matrix polarity are proposed and discussed. Quantitative barrier and mechanical data extracted from the reviewed studies are compiled and compared on a normalised basis, and the regulatory, safety, and scale-up barriers relevant to active pea protein-based packaging (EU food-contact-material compliance, migration testing, allergenicity, and life-cycle assessment) are discussed explicitly given the review’s focus on active food packaging. Overall, pea protein-based biodegradable films represent a promising and scientifically active alternative to petroleum-derived packaging materials, but their translation to commercial active-packaging products will depend on resolving moisture sensitivity, establishing standardised testing protocols, and generating the regulatory and techno-economic evidence base that is currently lacking.

## 1. Introduction

Food packaging is essential for maintaining product quality and minimising physical, chemical, and microbial deterioration during storage and distribution. However, the extensive use of petroleum-based plastics has raised major environmental and food safety concerns because of their persistence in the environment and potential migration of degradation products into foods. Consequently, biodegradable packaging materials derived from renewable resources have become a major focus of food-packaging research.

Natural polymers, particularly proteins and polysaccharides, have attracted considerable attention because of their biodegradability, biocompatibility, and renewability [1]. Among them, plant proteins represent promising alternatives for developing sustainable packaging materials. In recent years, pea-processing by-products have emerged as valuable feedstocks because they provide recoverable proteins and bioactive compounds while supporting circular-bioeconomy strategies [2].

**Figure 1:**
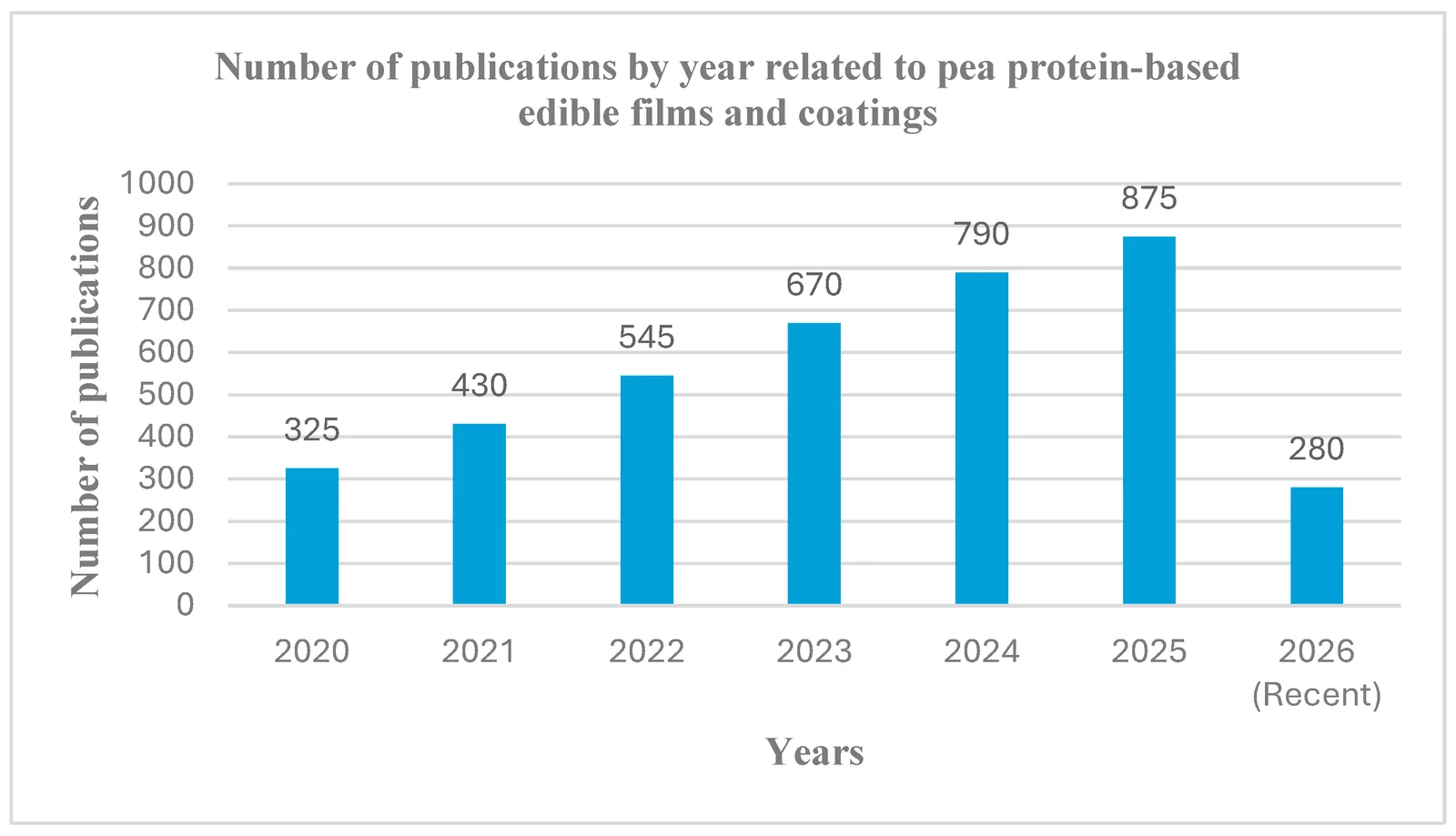
Number of publications by year related to pea protein-based edible films and coatings.

As shown in Figure 1, the scientific output related to pea protein films and their potential food-packaging applications has increased markedly over the past six years, reflecting the growing interest of the food-packaging community in this sustainable feedstock. The publication trend was generated using the Scopus database by searching the term “pea protein films” in the relevant publication records. The apparent decline observed for 2026 does not indicate a reduction in research activity but rather reflects that only a partial number of publications from 2026 had been indexed in Scopus at the time of the search. For the publication trend analysis, the Scopus database was searched using the query term “pea protein films”. The search covered publications indexed between 2020 and 2026.

The relevant literature was identified through structured Scopus searches (2015–2026, mainly 2020–2026) using combinations of the terms “pea protein”, “film”, “coating”, “active packaging”, “biodegradable”, “antimicrobial”, and “antioxidant” with Boolean operators. Studies were screened by title/abstract and full text, including only pea protein-based films, coatings, or composites for food-packaging applications. A total of 64 primary studies were synthesised, with selected related plant-protein or enzyme-modification studies included to support mechanistic interpretations.

Films produced solely from pea protein often exhibit limited mechanical strength and moisture resistance, restricting their commercial potential. Blending pea protein with biopolymers such as agar, chitosan, and gelatin improves structural integrity and functionality. The incorporation of antimicrobial and antioxidant agents further enables active-packaging applications. This review highlights recent advances in pea protein-based biodegradable antimicrobial films, focusing on extraction methods, fabrication strategies, physicochemical properties, bioactive performance, and challenges related to regulation and industrial-scale implementation.

Pea protein has emerged as a promising renewable biopolymer for sustainable packaging because of its biodegradability, low cost, and recovery potential from agricultural by-products, which helps address concerns related to petroleum-based packaging waste and microplastic pollution [3,4]. As a food-industry by-product, pea protein isolate (PPI) supports circular-bioeconomy principles while offering comparatively low allergenic potential relative to soy or wheat protein and suitability for food-contact applications. Because protein-based materials are naturally brittle, glycerol is commonly used as a plasticiser to improve flexibility and processability, enabling production through conventional methods such as extrusion and injection moulding.

### The Importance of Active Packaging

Conventional packaging mainly serves as a physical barrier and cannot actively control the factors responsible for food deterioration. In contrast, active packaging interacts with the packaged product to extend shelf life, improve safety, preserve quality, and maintain sensory attributes [5].

The growing importance of active packaging is associated with reducing food waste and enhancing food safety. By limiting oxidation and microbial contamination, these systems help minimise postharvest losses. Their key functions include oxygen and ethylene scavenging to delay oxidation and fruit ripening, as well as the controlled release of antimicrobial compounds that inhibit foodborne pathogens such as *Listeria monocytogenes* and *Escherichia coli* [6,7].

Edible films represent an advanced form of active packaging because they can be consumed together with food. Produced mainly from proteins and polysaccharides, these films reduce moisture loss and oxidative deterioration. Smart edible films can additionally provide real-time freshness monitoring through colour changes triggered by pH variations caused by spoilage metabolites [8]. Natural pigments such as anthocyanins and curcumin are widely used as indicators, although their stability remains a challenge.

## 2. Utilisation of Pea Waste and Protein Recovery

Pea (*Pisum sativum* L.) is cultivated on more than 25 million acres worldwide and has emerged as a sustainable feedstock for bioplastic production because of its low environmental impact and compatibility with circular-bioeconomy strategies. By-products generated during pea processing, including husks, defective seeds, and low-grade seeds, represent an abundant biomass resource containing cellulose, hemicellulose, starch, and residual proteins that can be valorised for biodegradable packaging applications [9].

Utilising these residues reduces production costs while supporting waste minimisation. Table 1 demonstrates that pea-derived materials cannot be considered as a single protein source because their functional performance is strongly determined by protein concentration, purification degree, and the presence of accompanying carbohydrates and fibres. Pea flour and protein concentrates provide economically attractive alternatives because they require less intensive processing and retain naturally occurring polysaccharides; however, their lower protein purity limits the formation of strong protein networks. In contrast, pea protein isolate (PPI) provides the highest protein density and is therefore the preferred fraction for biodegradable film applications due to its ability to form continuous protein-based matrices through intermolecular interactions. Processing residues, although limited in direct protein-film formation, represent valuable circular-bioeconomy resources because their polysaccharide fractions can contribute to reinforcement and composite film development [10,11,12].

Pea protein isolate (PPI) is considered a promising alternative to soy and wheat protein because of its comparatively hypoallergenic profile, balanced amino-acid composition, and protein purity of up to approximately 78–90% depending on the extraction route. The protein fraction consists mainly of albumins, globulins, and glutelins, with legumin (11S) and vicilin (7S) as the dominant storage proteins responsible for the material’s film-forming and emulsifying properties [15].

PPI exhibits functional characteristics suitable for active packaging, including high water- and oil-holding capacities, good emulsifying performance, and moderate thermal stability [8]. Its relatively high β-sheet content contributes to structural stability, whereas minimum solubility occurs near the isoelectric point (approximately pH 4–6), making pH control during processing an important variable. In addition, naturally occurring phenolic compounds—including ferulic acid, p-coumaric acid, and flavonoids—contribute antioxidant and antimicrobial activity, while the residual starch fraction improves gas-barrier performance [12].

### Extraction Methods

Pea protein can be extracted using conventional wet methods, dry physical fractionation, and several emerging green technologies. Conventional alkaline extraction followed by isoelectric precipitation remains the industrial standard because of its high purity, scalability, and low production cost [16]. However, exposure to alkaline conditions may induce protein denaturation, reduce functional properties, and generate large volumes of wastewater. Consequently, increasing attention has been directed toward sustainable extraction technologies that improve protein functionality while minimising environmental impacts.

**Figure 2:**
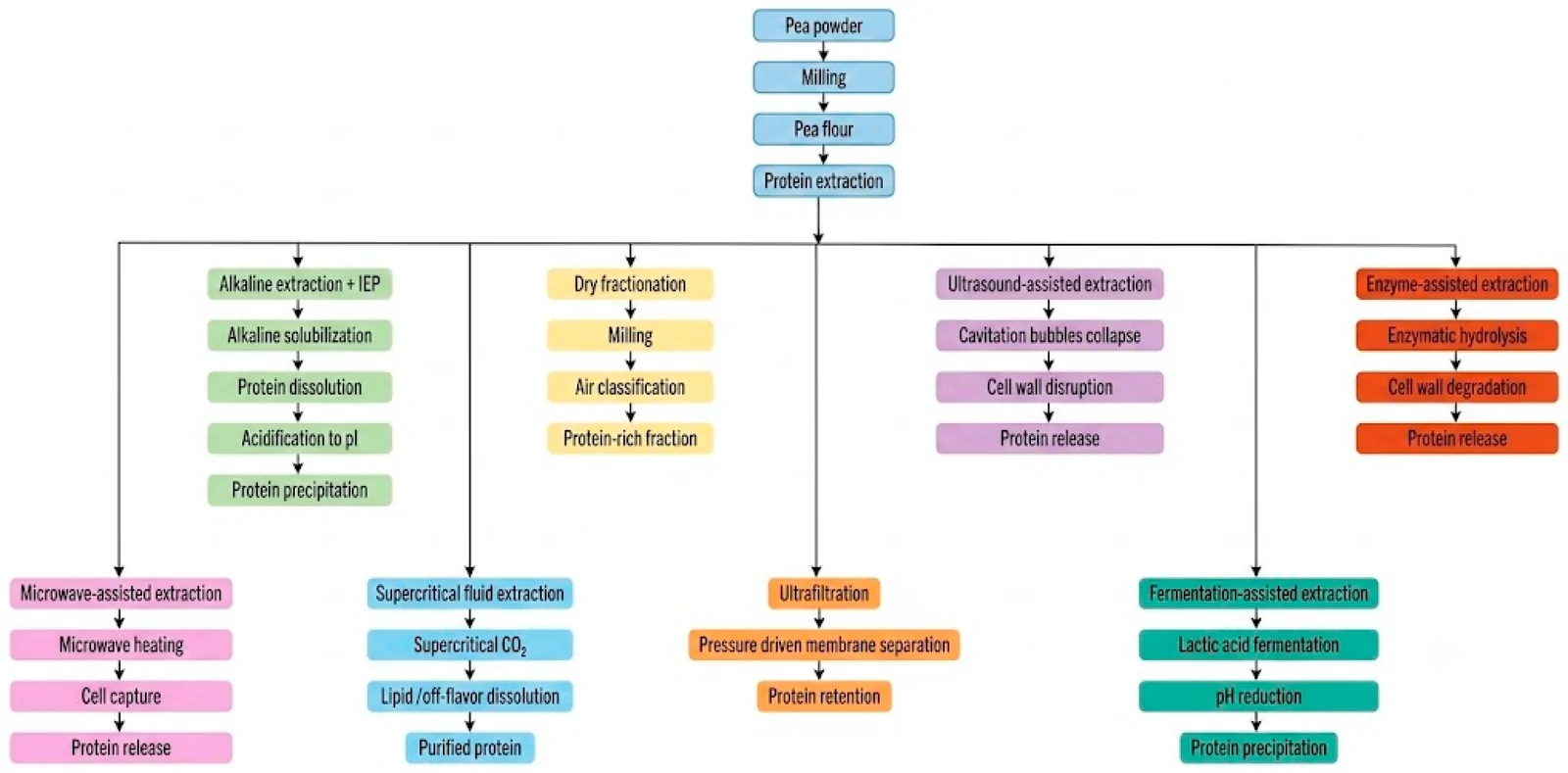
Schematic illustration of the principles of conventional and emerging pea protein extraction methods.

Emerging approaches, including ultrasound, enzyme, microwave and fermentation-assisted extraction, as well as ultrafiltration and supercritical fluid extraction, have demonstrated considerable potential for improving protein recovery, functionality, and sustainability. These techniques differ substantially in extraction mechanism, protein purity, processing time, environmental impact, and industrial applicability. Their major advantages and limitations are summarised in Table 2, while the extraction principles are illustrated in Figure 2.

As summarised in Table 2 and illustrated in Figure 2, the choice of extraction route involves a trade-off between protein purity and process cost, energy demand, and environmental impact, and this choice should itself be reported in Section 4 whenever the mechanical or barrier performance of a film is discussed, since extraction-induced denaturation directly affects the film-forming network described in Section 3.1.

Othmeni et al. [22] characterised field pea protein isolates in terms of colour, secondary structure, thermal properties, water and oil absorption, and pH-dependent solubility. The isolates were rich in β-sheet structures, denatured at approximately 83.8 °C, exhibited high water- and oil-holding capacities, and showed minimum solubility near the isoelectric point, supporting their suitability for biodegradable and active-packaging applications.

## 3. Film Production Strategies and Its Limitations

The formation of a film from pea protein is a complex process of molecular restructuring rather than a simple drying process.

### 3.1. Mechanism of Film Formation

Film formation in protein-based materials is governed by intermolecular interactions, including hydrogen bonding, hydrophobic interactions, ionic and dipole forces, and disulfide linkages. During processing, proteins are denatured by alkali, acids, heat, salts, or reducing agents, exposing hydrophobic and sulfhydryl groups that promote intermolecular association and network formation [2]. Under alkaline conditions (approximately pH 10–12), protein unfolding facilitates sulfhydryl/disulfide (SH/S–S) exchange reactions, increasing cross-link density during drying [23]. Plasticisers are subsequently incorporated to increase chain mobility and free volume, improving flexibility while reducing brittleness.

The final properties of pea protein isolate (PPI) films are therefore strongly determined by the processing conditions applied during extraction and film casting, including temperature, pH, ionic strength, and shear intensity. Moderate protein denaturation increases the availability of reactive functional groups and enhances intermolecular interactions, resulting in improved tensile strength and film integrity. However, excessive denaturation or uncontrolled aggregation before casting may reduce protein solubility, promote heterogeneous structures, and negatively affect mechanical performance. Thus, the relationship between extraction conditions, protein structure, and film properties represents an important consideration when comparing PPI films produced by different processing routes. Choi and Han [24] highlighted that heat-induced denaturation plays a key role in pea-protein-film formation through three consecutive stages: (i) denaturation of native protein structures and exposure of reactive sulfhydryl groups, (ii) formation of intermolecular interactions including disulfide bonds, hydrogen bonds, and hydrophobic associations, and (iii) consolidation of these interactions into a continuous three-dimensional polymer network during solvent evaporation.

Beyond physical interactions, enzymatic cross-linking using transglutaminase (TGase) provides an additional strategy to modify pea protein networks by catalysing the formation of ε-(γ-glutamyl)lysine covalent bonds between glutamine and lysine residues. This enzymatic reaction increases intermolecular cross-link density and can improve structural stability, water retention, and mechanical properties of pea protein matrices. Shen et al. [25] reported that TGase treatment increased the water-holding capacity of pea protein isolate from approximately 2.8 g/g protein in untreated samples to 5.2–5.6 g/g protein after cross-linking, indicating enhanced network hydration and structural compactness. Similarly, Yaputri et al. [26] demonstrated that TGase-induced polymerisation improved pea protein functionality by promoting protein aggregation and the development of stronger protein networks. However, the effect of TGase is highly dependent on enzyme concentration; moderate TGase levels (approximately 0.1–0.3%) can enhance network strength and structural stability, whereas excessive cross-linking may restrict molecular mobility, reduce flexibility, and negatively influence hydration and digestibility.

The composition of the biopolymer matrix is a major determinant of the functional performance of bio-based packaging films, influencing mechanical properties, flexibility, barrier performance, and thermal stability Individual protein-based matrices exhibit distinct advantages and limitations. Gelatin provides excellent film-forming ability and favourable oxygen and oil barrier properties; however, its hydrophilic nature limits water resistance and mechanical stability. Zein exhibits superior moisture-barrier performance due to its hydrophobic structure but tends to form brittle films, whereas soy protein isolate provides good oxygen-barrier properties but remains sensitive to moisture absorption [27].

To address these limitations, hybrid matrix strategies have been extensively investigated. Blending pea protein with polysaccharides or other proteins can improve film cohesion, mechanical performance, and functional properties. For example, chitosan incorporation enhances film-forming ability, thermal stability, and antimicrobial activity through electrostatic interactions with pea protein, whereas zein nanoparticles can reinforce pea protein isolate films by improving interfacial interactions and generating a denser polymer network [28]. In addition, cross-linking approaches combined with thermal treatment or pH modification provide further control over network density and mechanical properties of pea protein-based films [29].

Table 3 summarises matrix materials investigated in combination with pea protein according to their functional category (polysaccharides, proteins, and synthetic/biodegradable polyesters). The table distinguishes their typical role as blend components, coating/laminate layers, or structural materials where reported in the literature. Approximate matrix-to-pea protein ratios are included only when explicitly provided in the corresponding studies.

Pea protein films generally exhibit limited water resistance and reduced flexibility because of their hydrophilic nature and relatively weak intermolecular interactions compared with synthetic polymers. Therefore, composite matrix approaches, including polysaccharide, protein, and biodegradable polyester incorporation, are commonly employed to improve mechanical strength, barrier properties, and functional performance. As summarised in Table 3, chitosan and alginate-based systems have been investigated mainly as coating or blend components to enhance film cohesion, antimicrobial activity, and barrier properties, whereas gelatin can improve flexibility and compatibility within protein-based matrices [3,27].

### 3.2. Film Production Methods

Biopolymer-based films are produced using different laboratory- and industrial-scale techniques, each differing in processing conditions, scalability, energy demand, and their effects on the mechanical, barrier, and physicochemical properties of the final material.

- Solvent casting: Solvent casting is the most widely applied laboratory method for preparing protein-based films, including pea protein isolate. The protein is first dissolved under suitable pH conditions to improve solubility, and mild heat treatment (25–90 °C) is often applied to facilitate dissolution. The film-forming solution is then cast onto a flat surface and dried under controlled conditions to evaporate the solvent. This approach produces uniform, transparent films while allowing easy incorporation of plasticisers, lipids, proteins, and bioactive compounds such as antimicrobial and antioxidant agents [34]. Film thickness can also be accurately controlled using coating blades. However, prolonged drying and batch processing limit its industrial applicability. Acquah et al. [35] prepared films by dissolving yellow pea protein isolate (YPI), whey protein isolate (WPI), protein concentrate, and YPI–WPI (1:1) blends in deionised water (4%, *w*/*v*). The pH was adjusted to 8.0 with 0.1 M NaOH, followed by heating at 75 °C for 30 min to denature the proteins. Glycerol (50%, *w*/*w*) was incorporated as a plasticiser, the mixture was stirred for an additional 30 min, cast into Petri dishes, dried at 25 °C for 72 h, and conditioned before characterisation.
- Extrusion: Extrusion is the preferred technique for industrial production because it supports continuous processing and large-scale manufacturing. The process includes feeding, compression under increasing temperature and pressure, and die shaping, where the material is subjected to elevated temperature, pressure, and shear under low-moisture conditions. Although these conditions enable rapid and efficient production, heat-sensitive biopolymers require precise control of processing parameters to minimise thermal degradation. Screw design, temperature profile, and residence time are therefore critical variables. Plasticisers improve melt processability, whereas twin-screw extruders provide superior mixing and material homogenisation. Compared with solvent casting, extrusion requires less water and offers higher productivity. High-moisture extrusion has also been reported to improve film flexibility and functionality [36].

**Figure 3:**
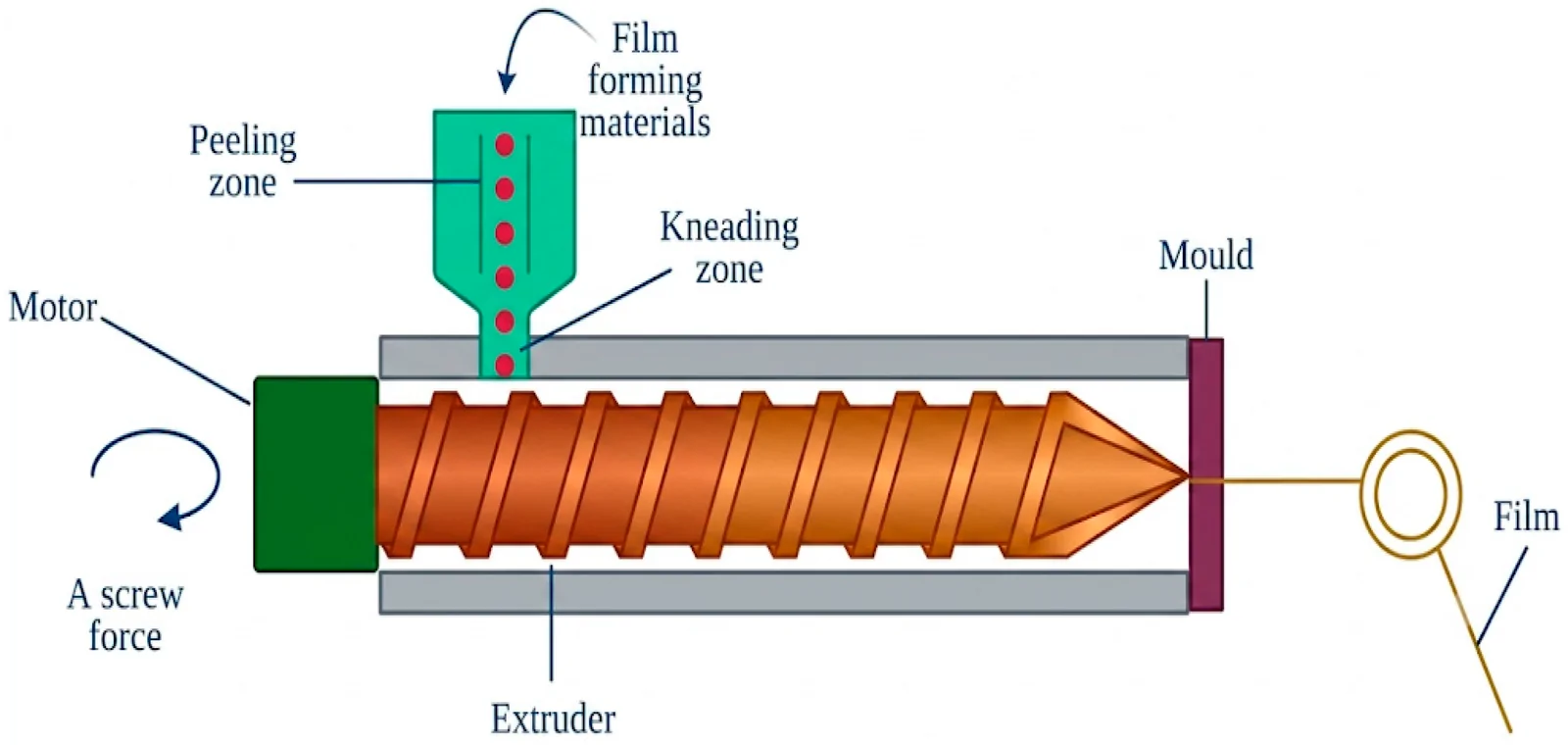
Schematic representation of the extrusion method.

Figure 3 depicts the feeding, kneading, and die (mould) zones of a typical single-screw extruder configuration used for protein-based film production.

- Blown-film extrusion: Blown-film extrusion is an industrial thermomechanical process in which molten biopolymer is extruded through an annular die, expanded by internal air pressure, and cooled to form a film. The biaxial orientation generated during bubble inflation enhances mechanical strength and barrier performance compared with cast films. However, the low melt strength of many biopolymers may reduce bubble stability, which can be improved by incorporating long-chain branching agents. Huntrakul et al. [31] produced films containing acetylated cassava starch and pea protein isolate using a two-step extrusion process. Mixtures containing 5%, 10%, and 20% pea protein isolate were compounded with glycerol and water in a twin-screw extruder at temperatures up to 160 °C to produce pellets, which were subsequently processed into films using a single-screw blown-film extruder. Pea protein reduced the stickiness of starch-based formulations, enhanced bubble stability, and enabled the production of homogeneous edible films suitable for industrial-scale manufacture.
- Electrospinning: Electrospinning is an electrohydrodynamic technique that generates micro- and nanofibers by applying a high electric field to a polymer solution, producing a Taylor cone followed by solvent evaporation. The resulting fibrous structures possess a high surface-area-to-volume ratio, which makes this method particularly attractive for active and functional food packaging. Although this same open fibrous structure is inherently highly porous, which increases gas permeability and can be detrimental to oxygen-sensitive food applications unless the fibre mat is combined with a denser barrier layer.

**Figure 4:**
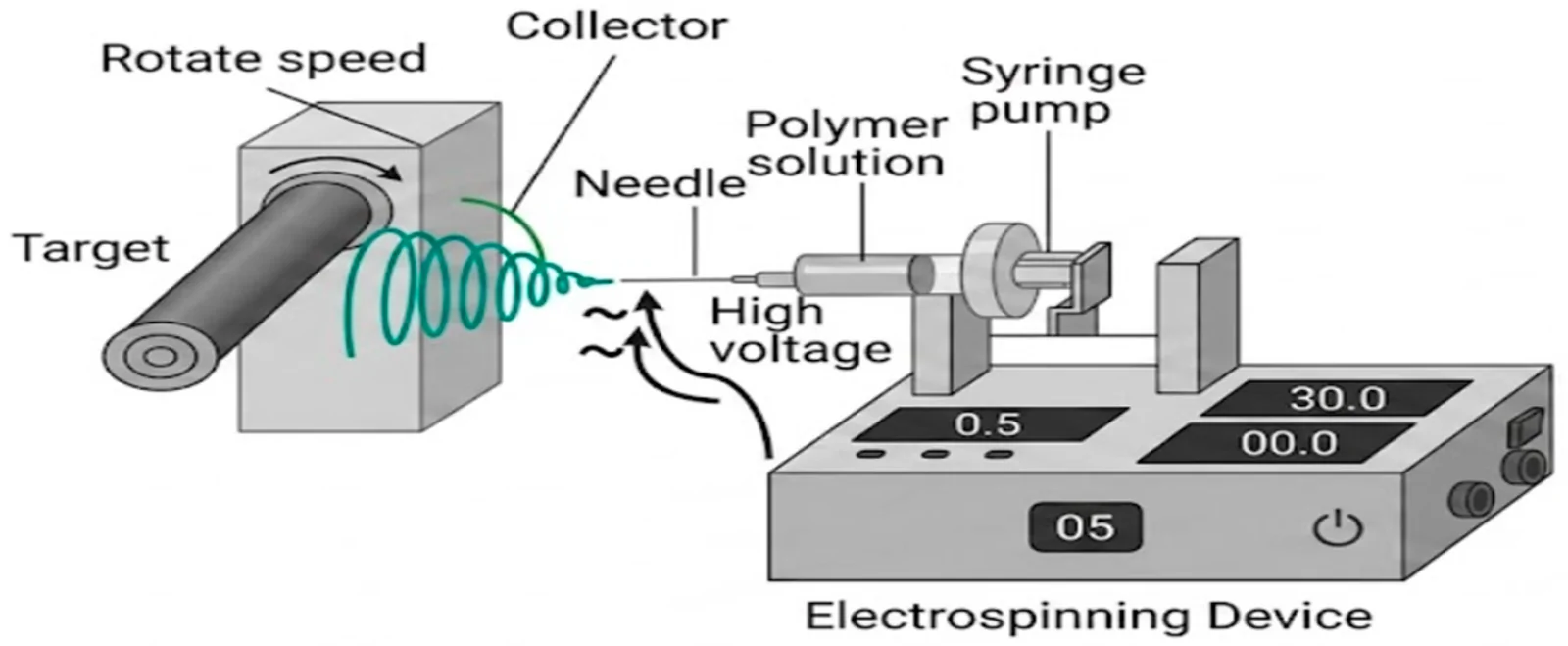
A schematic demonstration of electrospinning process.

As illustrated in Figure 4, the technique relies on a high-voltage field between the syringe needle and a rotating collector to draw the polymer solution into continuous nanofibres.Because pea protein alone exhibits limited spinnability, it is commonly blended with electrospinnable polymers such as pullulan, polyethylene oxide (PEO), or polyvinyl alcohol (PVA), which improve viscosity and molecular-chain entanglement. Jiang et al. [37] reported that partial denaturation of pea protein and increased solution conductivity under acidic conditions were essential for stable fibre formation; optimum electrospinning was achieved at 20–26 kV with solution viscosities of 0.9–1.6 Pa·s, producing uniform fibres with diameters between 180 and 520 nm, and relative humidity below 35% increased the risk of needle-tip clogging. Najafi et al. [38] produced electrospun nanofibres by blending pea protein isolate with pullulan and pectin; the nanofibres exhibited a uniform, bead-free morphology, CaCl_2_ cross-linking improved water resistance, and release was governed predominantly by Fickian diffusion, highlighting their potential as bioactive-delivery systems for food packaging (see Section 5.3 for a fuller discussion of release kinetics).

- Multilayer Films: Multilayer films are designed to overcome the limited mechanical and barrier performance of biopolymers by integrating layers with distinct functions, such as gas and moisture barriers or mechanical reinforcement. These structures are produced using lamination, extrusion, or co-extrusion, and their performance can be further enhanced by incorporating nanomaterials or synthetic polymer coatings [6].

### 3.3. Food Film Coating Methods

Coating and wrapping techniques play a critical role in determining the uniformity, thickness, adhesion, and protective performance of edible films and coatings. The selection of the application method depends on the characteristics of the food product, coating formulation, production scale, and desired barrier properties [31]. Conventional application techniques include wrapping, immersion, spray coating, fluidised bed coating, and pan coating. Each method differs in its operating principle, coating uniformity, coating thickness, industrial applicability, and limitations. Immersion and spray coating are the most commonly employed for fresh fruit and vegetables because of their simplicity and cost-effectiveness, whereas fluidised-bed and pan coating are preferred for particulate products requiring highly uniform coatings. Wrapping differs from the other techniques because the film is produced separately and subsequently applied to the food surface. Table 4 summarises the main characteristics, advantages, limitations, and typical applications of these methods.

## 4. The Physicochemical and Functional Properties of Films

The functional performance of pea protein-based films is governed by the interactions among protein chains, plasticisers, and incorporated bioactive compounds. Protein functional groups, particularly amino, carboxyl, sulfhydryl, and hydroxyl-containing residues, contribute to intermolecular hydrogen bonding and network formation [21]. 

Plasticisers such as glycerol and sorbitol modify these interactions by increasing polymer-chain mobility; consequently, they generally decrease tensile strength while improving flexibility and elongation at break. However, the magnitude of these effects depends on plasticiser molecular characteristics, concentration, and compatibility with the protein matrix.

Previous studies have frequently reported the effects of individual additives independently, making direct comparison difficult because differences in protein concentration, plasticiser type, additive loading, and testing conditions can strongly influence film performance. Therefore, the present review integrates reported mechanical and barrier properties and discusses possible structure–property relationships underlying the observed trends. The mechanisms proposed below should be considered plausible interpretations based on established protein-film chemistry rather than experimentally verified explanations unless directly demonstrated by the original studies.

### 4.1. Mechanical Performance

Acquah et al. [35] determined the thickness of yellow pea protein-based films at multiple locations using a digital calliper and evaluated mechanical properties with an Instron universal testing machine. Film thickness was uniform across the surface. Whey protein isolate (WPI) films showed the highest tensile strength and stiffness, whereas yellow pea protein isolate (YPI) films exhibited superior elongation at break. Yellow pea protein concentrate films displayed lower mechanical strength because reduced protein content limited intermolecular hydrogen bonding and increased molecular mobility, consistent with the protein-source comparison in Table 1 (Section 3.1), where concentrate-grade materials formed less dense networks than isolates. Huntrakul et al. [31] characterised cassava starch/pea protein films produced by blown-film extrusion according to ASTM D882 [39]. Tensile strength (TS) and elongation at break (EB) were measured in machine (MD) and cross directions (CD), while DMA analysis (0.5–20 Hz, 100–120 °C) demonstrated that processing conditions and matrix composition regulate mechanical and viscoelastic behaviour.

Ayazi et al. [40] measured films containing pea protein, sage seed gum, and cumin essential oil using a digital micrometre and ASTM D882. Increasing PPI content from 50% to 75% significantly increased thickness, tensile strength, and elastic modulus (*p* < 0.05), supporting the network-density mechanism discussed above. Conversely, higher cumin-essential-oil levels reduced tensile strength but improved flexibility, consistent with a plasticising effect. As summarised in Table 5, Cheng et al. [41] reported that garlic oil caused no significant mechanical changes in PPI films, whereas ginger and particularly cinnamon oil reduced tensile strength and elongation at break. Cheng et al. [42] similarly observed that increasing thyme-oil concentration in nano- or microemulsions reduced elongation at break (minimum 39.44% at 2%). Collectively, these studies indicate that mechanical performance depends not only on essential-oil concentration but also on the polarity and reactivity of dominant compounds (e.g., organosulfur compounds in garlic oil versus phenolic aldehydes in cinnamon oil). This structure–property relationship has rarely been explicitly discussed in pea-protein-film literature and represents a key insight of this review.

Maia et al. [43] evaluated pea protein isolate/tannic-acid films after conditioning at 22 °C and 50% relative humidity for 48 h according to ASTM D882. Tannic acid increased tensile strength from 3.86 to 6.45 MPa through intermolecular cross-linking, although elongation decreased due to network stiffening. Cheng et al. [28] reported that eugenol-loaded zein nanoparticles increased tensile strength of pea starch/soy protein isolate films approximately 1.7-fold at 3% loading while reducing elongation, indicating nanocomposite reinforcement rather than hydrogen-bond-driven strengthening (Table 5). Nan et al. [44] and Alshabrmi et al. [33] identified optimum polyphenol concentrations, with maximum tensile strength at 4% Aronia polyphenols and 8% green tea polyphenols, respectively, followed by declines at higher levels. This concentration-dependent trend across tannic acid, green tea catechins, and Aronia polyphenols suggests a two-stage mechanism: moderate phenolic addition promotes hydrogen bonding and cross-linking with protein amide groups, whereas excessive loading may increase water competition, act as an internal plasticiser, or disrupt protein aggregation. However, these structural changes were not directly characterised by techniques such as circular dichroism or amide-I FTIR deconvolution and require further investigation.

Faust et al. [36] examined plasticiser effects in pea protein isolate films produced by high-moisture extrusion. Glycerol reduced intermolecular interactions, decreasing tensile strength but increasing ductility; films with 20% glycerol showed approximately 8 MPa tensile strength, while 60% glycerol increased elongation at break to 102%. In contrast, sorbitol produced stiffer and more brittle films due to its higher molecular weight and lower plasticising efficiency, confirming that plasticiser selection strongly influences mechanical performance independent of other additives.

### 4.2. Barrier Performance: Oxygen and Water-Vapour Permeability

#### 4.2.1. Water-Vapour Permeability

Acquah et al. [35] evaluated the surface hydrophobicity of yellow pea protein-based films using the sessile-drop method, measuring the contact angle of a 2 μL distilled-water droplet within 10 s (n = 10 independent measurements). Immersion experiments showed that part of the film dissolved in water, likely because of loosely bound plasticiser or protein fractions not fully incorporated into the matrix; moisture content increased approximately 42-fold in whey protein isolate (WPI) films and 27-fold in yellow pea protein isolate (YPI) films after immersion, although all films retained structural integrity. Ayazi et al. [40] determined moisture content by drying samples at 105 °C for 24 h, water solubility after immersion in 50 mL distilled water for 6 h, and swelling ratio after immersion in 30 mL distilled water until constant weight. Increasing cumin-essential-oil content significantly reduced (*p* < 0.05) moisture content, water solubility, and swelling because of its hydrophobic nature, whereas higher sage-seed-gum content increased these parameters because of its hydrophilic character.

Cheng et al. [42] measured water-vapour permeability of thyme-oil-loaded pea protein films according to ASTM D882-95 [45]; permeability increased when thyme oil exceeded 1.0% and, at 2.0%, nanoemulsion-based films exhibited superior moisture-barrier performance to microemulsion systems (Table 5). Maia et al. [43] evaluated WVP according to ASTM E96/E96M [46] using CaCl_2_-filled permeability cells at 25 °C and 75% relative humidity, finding an 87.9% reduction in WVP for films containing 10% tannic acid. Alshabrmi et al. [33] determined WVP gravimetrically using CaCl_2_-filled cups at 75% relative humidity, and Faust et al. [36] found that sorbitol promoted microcrack formation during drying, resulting in higher WVP and oxygen permeability than glycerol, while glycerol increased water retention because of its hygroscopic nature.

#### 4.2.2. Oxygen and Light Barrier

Although pea protein-based films are frequently considered promising oxygen-barrier materials because of their dense protein networks, quantitative oxygen permeability values remain limited among the reviewed studies. Therefore, statements describing pea protein films as having “excellent oxygen-barrier properties” should be avoided unless supported by direct oxygen permeability measurements.

Among the reviewed studies, Faust et al. [36] specifically examined oxygen permeability and demonstrated that plasticiser type influenced oxygen transport behaviour. Sorbitol-containing films exhibited higher oxygen permeability than glycerol-containing films, which was attributed to structural defects generated during drying. This finding highlights that oxygen transport is strongly affected by film microstructure and plasticiser-induced changes in polymer mobility.

### 4.3. Optical Properties

Ayazi et al. [40] analysed the optical properties of pea protein/sage-seed-gum films over the 200–800 nm wavelength range and reported that increasing sage-seed-gum content improved transparency, whereas cumin essential oil reduced light transmission. This reduction may be associated with increased light scattering caused by dispersed oil phases within the film matrix rather than representing a general transparency-modifying effect of natural additives. Cheng et al. [41] observed that essential oils did not significantly alter opacity at the tested concentrations, suggesting that the optical effect of essential oils depends on their concentration, chemical composition, and dispersion behaviour.

Similarly, Maia et al. [43], Cheng et al. [28], and Alshabrmi et al. [33] reported reduced light transmission and enhanced UV-shielding capacity with increasing phenolic compound or nanoparticle loading. These effects may be explained by several possible mechanisms, including (i) molecular absorption, where chromophoric groups present in phenolic compounds or other bioactive additives absorb specific UV–visible wavelengths; (ii) changes in film homogeneity, where improved dispersion promotes uniform light transmission while aggregated domains or phase separation increase scattering; and (iii) physical light scattering caused by nanoparticles, undissolved phenolic compounds, or dispersed droplets within the film matrix.

### 4.4. Colour Analysis

Acquah et al. [35] found that film colour was significantly affected by both protein source and the pH of the film-forming solution before plasticiser addition. Ayazi et al. [40] reported that cumin essential oil significantly decreased both lightness (L) and whiteness index in PPI–sage-seed-gum films, and Cheng et al. [41] found that garlic and cinnamon oils reduced lightness while increasing redness, with all tested oils increasing yellowness (b), most strongly with cinnamon oil—consistent with the greater chromophoric/reactive character of cinnamaldehyde. Maia et al. [43], Cheng et al. [28], and Alshabrmi et al. [33] similarly reported progressive darkening and increased total colour difference (ΔE) with increasing phenolic or nanoparticle content, confirming that colour change is a broadly consistent, concentration-dependent indicator of additive incorporation across the systems reviewed.

### 4.5. Structural Analysis

Acquah et al. [35] characterised the morphology, molecular structure, and thermal behaviour of yellow pea protein-based films using SEM, FTIR, and DSC. SEM images revealed particle aggregates in the film surface, and DSC analysis (−40 to 300 °C, 10 °C/min, N_2_ atmosphere) showed two endothermic transitions corresponding to glass transition and melting, confirming the partially amorphous nature of the films. SEM images revealed particle aggregates and a heterogeneous surface, and DSC analysis showed two endothermic transitions consistent with a glass transition and a melting/denaturation event, indicating a partially amorphous film structure; whether the observed aggregation is causally related to the particle-size distribution of the casting solution was not established by the cited data and should not be asserted without direct particle-size measurement.

Huntrakul et al. [31] examined cassava starch/pea protein films using SEM, FTIR, XRD, DSC, and TGA; XRD (4–40° 2θ) determined crystallinity, and TGA (25–800 °C) evaluated degradation behaviour, showing that polymer composition governs the crystalline structure and thermal stability of the starch–protein films. Ayazi et al. [40] reported that FTIR O-H band intensity decreased with increasing essential-oil concentration (indicating stronger intermolecular interaction) and that XRD-confirmed crystallinity increased with blend and essential-oil content, alongside higher decomposition temperatures at 2% essential oil. Cheng et al. [41] observed by SEM that neat pea protein films had a dense, layered morphology, whereas essential-oil incorporation increased pore formation and structural heterogeneity, with FTIR indicating new hydrogen bonds between essential oils and the protein matrix accompanied by weakened protein–protein interaction a structural observation consistent with the plasticising mechanism proposed for cinnamon oil in Table 5. Maia et al. [43] found, by FTIR, XRD, and TGA, that tannic acid produced only minor changes in amide bands and limited improvement in thermal stability indicating that mechanical and thermal reinforcement do not necessarily track together in these systems. Cheng et al. [28] and Nan et al. [44] both reported reduced crystallinity with increasing nanoparticle or polyphenol loading, together with FTIR evidence of stronger hydrogen bonding, reinforcing the two-regime optimum-loading mechanism proposed in Section 5.1.

## 5. The Bioactive Functions of Films

Pea and other plant proteins are effective carriers for the controlled release of bioactive compounds in active-packaging systems. Essential oils (e.g., thyme and cinnamon) and nanoparticles incorporated into the protein matrix maintain higher concentrations of active agents at the food surface, thereby suppressing microbial growth [47]. However, the effectiveness of bioactive films depends not only on the concentration of active compounds but also on their chemical structure, interaction with the protein matrix, dispersion state, and release behaviour.

Essential oils, plant extracts, and phenolic compounds can provide antimicrobial and antioxidant functions, while their incorporation may simultaneously modify mechanical and barrier properties through interactions with protein chains. Nevertheless, the functional performance of these films should be interpreted considering both the activity of the bioactive compound itself and its controlled availability within the polymer network.

### 5.1. Antimicrobiyal Analyses

Ayazi et al. [40] evaluated the antimicrobial activity of pea protein-based films containing sage-seed extract and cumin essential oil (CEO). Films without CEO showed no inhibition against *Escherichia coli*, *Staphylococcus aureus*, *Candida albicans*, or *Aspergillus niger*, whereas CEO incorporation significantly enhanced antimicrobial activity (*p* < 0.05). The strongest inhibition was observed against *S. aureus*, indicating that essential-oil incorporation substantially improved the antimicrobial functionality of the protein matrix.

Cheng et al. [41] investigated the effects of different essential oils incorporated into pea protein isolate (PPI) films. Garlic oil exhibited the strongest activity against *S. aureus* but showed limited effects against *S. typhimurium* and *E. coli*, whereas cinnamon oil demonstrated broader antimicrobial activity, particularly against Gram-negative bacteria. Cheng et al. [42] reported inhibition zones of 23.00–27.80 mm against three *Salmonella* strains for films containing 2.0% thyme oil, with nanoencapsulation further improving antimicrobial performance. Maia et al. [43] observed that tannic-acid-containing films significantly inhibited *S. aureus* but showed limited activity against *E. coli*. Similarly, Alshabrmi et al. [33] reported inhibition rates of 94.33% for *S. aureus* and 90.99% for *E. coli* at 12% green-tea-polyphenol loading.

### 5.2. Analysis of Antioxidant Capacity

Antioxidant activity is an important functional property of active-packaging films because oxidative reactions contribute to quality deterioration of packaged foods. However, antioxidant capacity values obtained from different analytical assays should not be directly compared because each method measures different chemical mechanisms. Ayazi et al. [40] evaluated antioxidant activity using DPPH radical-scavenging activity measured at 517 nm and total phenolic content using the Folin–Ciocalteu method at 765 nm with gallic acid as standard. The incorporation of cumin essential oil increased both DPPH activity and phenolic content, demonstrating improved antioxidant functionality.

Maia et al. [43] reported 61% DPPH radical-scavenging activity for films containing 10% tannic acid. Cheng et al. [28] demonstrated that eugenol-loaded nanoparticles improved antioxidant activity using the DPPH assay. Nan et al. [44] evaluated *Aronia melanocarpa* polyphenol-containing films using DPPH, ABTS, and FRAP assays and reported enhanced antioxidant potential compared with the control system. Alshabrmi et al. [33] found that increasing green-tea-polyphenol loading from 0 to 12% increased DPPH antioxidant activity from 20.90% to 58.37%.

### 5.3. Controlled Release: Kinetics and Limitations

Controlled release of antimicrobial and antioxidant compounds from the film matrix is essential for maintaining long-term active-packaging performance. Release behaviour depends on diffusion, polymer relaxation, swelling, compound solubility, and interactions between active molecules and protein chains. Bioactive release from pea protein-based matrices is commonly described using Fickian diffusion models, where molecular migration is governed by concentration gradients and diffusion coefficients. Early-stage release generally follows a square-root-of-time relationship, while the diffusion coefficient is affected by temperature, polymer free volume, swelling, and molecular interactions. However, Fickian models are limited to initial release stages because diffusion slows as concentration gradients decrease. Moreover, finite bioactive content may lead to depletion during storage, reducing antimicrobial or antioxidant activity despite structural stability of the film. Although several studies have demonstrated antimicrobial and antioxidant effects of pea protein-based films [28,33,40,43], most evaluations were conducted over relatively short periods, and extended release profiles under realistic food-storage conditions remain limited.

### 5.4. Biodegradable Analyses

Biodegradability is an important advantage of protein-based packaging materials; however, degradation behaviour is strongly dependent on environmental conditions and testing methodology. Ayazi et al. [40] evaluated biodegradability of pea protein isolate/sage-seed-gum/cumin-essential-oil films using a soil-burial test. Film samples were buried in soil for 60 days, periodically removed, and analysed based on weight loss. More than 90% biodegradation was reported, with near-complete decomposition at the end of the test period. Higher sage-seed-gum content accelerated degradation, whereas increased pea protein content slowed the process, indicating that polymer composition influences biodegradation behaviour.

## 6. Food Application of Pea Protein-Based Films

The practical performance of pea protein-based active films depends on the compatibility between bioactive compounds and the dominant deterioration mechanisms of target foods. Essential oils, polyphenols, and nanoparticles provide antimicrobial, antioxidant, and barrier functions; however, their effectiveness is influenced by compound chemistry, release behaviour, food composition, and storage conditions. Application studies are therefore essential to determine whether laboratory findings translate into real food systems.

Huntrakul et al. [31] developed cassava starch/pea protein films by blown-film extrusion and produced sachets containing soybean or olive oil. After 3 months of storage under dark conditions at 30% and 50% relative humidity, both starch and pea protein sachets reduced lipid oxidation. However, starch-only sachets showed greater shrinkage and deformation, while pea protein improved dimensional stability and UV protection, contributing to antioxidant preservation and physical integrity. Cheng et al. [41] evaluated pea protein isolate films containing garlic, ginger, and cinnamon essential oils for salmon preservation. While pure PPI films lacked antibacterial activity, films containing 2% essential oils inhibited microbial growth, with cinnamon oil showing stronger activity than garlic oil. This highlights the importance of selecting essential oils based on target microorganisms and active compounds rather than concentration alone.

Cheng et al. [42] applied pea protein films containing oregano essential oil (OEO) to chicken breast during refrigerated storage. Films with 2.0% OEO reduced aerobic plate counts by 33.28% and extended shelf life by approximately 5 days, likely due to sustained release of antimicrobial compounds from the protein matrix. Cheng et al. [28] investigated pea starch/soy protein isolate films containing eugenol-loaded zein nanoparticles for strawberry preservation. Nanoparticle-containing films prevented mould growth during storage and reduced weight loss from over 38% to 24% in the most effective formulation (PSZ3), indicating improved preservation through antimicrobial activity, antioxidant effects, and modified moisture transfer.

Nan et al. [44] developed pea protein–chitosan films enriched with *Aronia melanocarpa* polyphenols, showing strong antioxidant activity, particularly under acidic conditions. Their effectiveness depends on polyphenol stability and release behaviour within food environments. Alshabrmi et al. [33] applied pea protein/corn starch films containing green-tea polyphenols to beef during refrigerated storage. These films delayed microbial growth, protein degradation, and lipid oxidation while maintaining colour, water-holding capacity, and sensory quality. The effects were attributed to reduced oxygen permeability and sustained polyphenol availability; however, differences among antioxidant assays require cautious comparison across studies.

## 7. Discussion

The reviewed studies demonstrate that proteins recovered from pea-processing by-products represent promising raw materials for the development of biodegradable and active food-packaging films. The incorporation of pea proteins into composite matrices with other biopolymers can improve mechanical performance and barrier functionality, while essential oils, polyphenols, and nanoparticle-based additives provide additional antimicrobial and antioxidant functions. However, the reported improvements are not universally proportional to additive concentration. As demonstrated in Table 5 and the mechanistic discussion sections, particularly for polyphenol-containing systems, excessive additive loading may negatively affect film performance by increasing hydrophilicity, disturbing polymer-chain organisation, or reducing structural homogeneity. Therefore, formulation optimisation should focus on identifying concentration thresholds that provide maximum functional benefits rather than assuming that increased additive content will always improve performance.

Food-application studies indicate that pea protein-based active films can improve food quality by reducing oxidation, microbial growth, and moisture-related deterioration when the selected active compound is matched with the dominant spoilage mechanism of the target food. Antimicrobial essential oils are particularly relevant for high-moisture foods such as seafood and poultry, whereas polyphenol-containing systems may provide additional benefits for lipid-rich foods susceptible to oxidation. Nevertheless, successful laboratory-scale preservation does not automatically translate into commercial application, and further validation under realistic storage, distribution, and consumer-use conditions is required.

Economic feasibility and supply-chain availability are also critical factors for commercialisation. Although pea-processing by-products represent attractive sustainable resources, consistent industrial supply of suitable protein fractions, efficient extraction technologies, and cost-effective processing routes remain important considerations. Life-cycle assessment (LCA) and techno-economic analysis should therefore accompany material development to determine whether environmental benefits are maintained after considering extraction, purification, processing energy, and end-of-life management.

Because these materials are intended for active food-packaging applications, regulatory and safety considerations require further attention. Commercial implementation requires compliance with food-contact-material regulations, including migration assessment of incorporated essential oils, polyphenols, nanoparticles, and other active components. For applications in the European market, evaluation under the EU Food Contact Materials regulatory framework is required, including assessment of overall migration and specific migration limits where applicable. In addition, although pea proteins are generally considered safe food ingredients, potential allergenic concerns associated with pea proteins should be considered, particularly for consumers with legume-related sensitivities. Future studies should therefore include migration testing, toxicological evaluation, allergenicity assessment, and regulatory risk analysis alongside functional performance evaluation.

## 8. Conclusions

Proteins recovered from pea-processing by-products represent promising renewable resources for biodegradable, active food-packaging materials, and this review has synthesised current evidence on pea protein sources, extraction routes, film-formation mechanisms, processing technologies, physicochemical properties, bioactive functionality, and food applications across different biopolymer matrices. A recurring, cross-cutting finding is that additive performance is concentration-dependent rather than linear: for polyphenols, essential oils, and nanoparticles alike, moderate loading generally improves mechanical and barrier properties, while excessive loading increases hydrophilicity or disrupts the polymer network and can reverse these gains (Table 5). Several barriers must still be resolved before commercial translation is feasible. Moisture sensitivity remains the primary limitation and should be evaluated against application-specific benchmarks (e.g., ASTM E96 [46]/ ASTM D570 [48] or ISO 62 [49]/ ISO 2528 [50]) rather than judged in absolute terms. Controlled-release behaviour is currently characterised mainly over short experimental periods, and long-term migration and repeated microbial-challenge studies are needed to confirm that antimicrobial and antioxidant protection is maintained across realistic shelf lives. Biodegradation claims, most notably the >90% soil-burial weight loss reported for pea protein isolate/sage-seed-gum films, should not be generalised without standardised testing (ISO 14855-1 [51], ISO 17556 [52]) and characterisation of degradation intermediates. Translation to industrial scale will additionally require attention to raw-material and processing variability, life-cycle and techno-economic assessment, and regulatory compliance, including EU food-contact-material migration testing and allergenicity evaluation for legume proteins. Advances in enzymatic modification, nanotechnology, and formulation-screening tools may accelerate this development, but realising commercially viable pea protein active packaging will ultimately depend on coordinated efforts among material scientists, food technologists, regulators, and industry across the circular-bioeconomy value chain.

## Figures and Tables

**Table 1:** Comparison of pea-processing protein sources and their relevance to film formation.

Protein Source	Typical Protein Content	Main Co-Constituents	Processing Level and Characteristics	Relevance to Film Formation	References
**Pea flour**	~20–25%	Starch, fibre, residual lipids, dietary fibre, minerals	Least-processed pea fraction obtained by milling whole peas; retains most native components	Starch co-matrix can act as an internal plasticiser/filler but dilutes protein network density, generally lowering tensile strength relative to isolate-based films	[10,11]
**Pea protein concentrate**	~55–70%	Residual carbohydrates, fibre, minor lipids	Intermediate fraction produced by partial removal of starch and soluble components	Intermediate network density; residual carbohydrate can compete for water and affect plasticiser distribution	[11,13]
**Pea protein isolate (PPI)**	~80–90%	Minor lipids, phenolics, antinutritional factors	Highly purified protein fraction obtained mainly by alkaline extraction/isoelectric precipitation or alternative fractionation technologies	Highest protein network density; the dominant substrate in the mechanical/barrier studies reviewed in Section 6	[12,14]
**Processing residues (husk, defective-seed fraction)**	Variable, typically <20% on a dry-weight basis	Cellulose, hemicellulose, lignin-like fractions, starch	Agricultural/industrial by-products generated during pea processing; require valorisation strategies	Mainly a source for co-extracted polysaccharide reinforcement rather than the principal film-forming protein	[11,14]

**Table 2:** Comparison of conventional and emerging pea protein extraction methods.

Extraction Method	Principle	Protein Purity/Yield	Advantages	Limitations	Reference
**Alkaline extraction + isoelectric precipitation**	Solubilisation under alkaline pH followed by precipitation at pI	80–90%	Industrial standard, inexpensive, scalable	Protein denaturation, wastewater generation	[3]
**Dry fractionation**	Air classification based on particle size and density	Moderate	Water-free, low energy, native protein preserved	Lower purity	[17]
**Ultrasound-assisted**	Acoustic cavitation disrupts cell walls	Yield up to 25%	Short extraction time, improved solubility	Aggregation under high power	[18]
**Enzyme-assisted**	Enzymatic hydrolysis of cell wall polysaccharides	90–95%	Highest purity, tailored functionality	High enzyme cost	[14]
**Microwave-assisted**	Rapid dielectric heating	High	Fast extraction, reduced solvent	Overheating, Maillard reaction	[17]
**Supercritical fluid extraction**	Supercritical CO_2_ selectively removes lipids/off-flavours	>90%	Excellent flavour improvement, solvent-free	High equipment cost	[19,20]
**Ultrafiltration**	Pressure-driven membrane separation	70–80%	Excellent functional properties	Membrane fouling	[14]
**Fermentation-assisted**	Microbial acidification and biological modification	70–75%	Improved flavour, antioxidant activity	Long processing time	[21]

**Table 3:** Classification of matrix materİals used with pea protein in biodegradable films (polysaccharides, proteins, and synthetic/biodegradable polyesters), with their functional role relative to pea protein.

Class/Matrix Material	Main Advantages	Main Limitations	Role Relative to Pea Protein	References
**Polysaccharide-** **Agar**	Excellent gel formation, transparency, good mechanical strength, edible, low oxygen permeability	Moisture sensitive, brittle without plasticiser, high water vapour permeability	Blend component; improves film strength and structural integrity	[30]
**Polysaccharide-** **Alginate**	Biocompatible, biodegradable, calcium-induced gelation, transparent	High water vapour permeability, moisture sensitive, limited thermal stability	Blend component; enhances gel network and O_2_ barrier	[31]
**Polysaccharide-** **Chitosan**	Natural antimicrobial activity, biodegradable, biocompatible, electrostatic interaction with proteins	Brittle, limited water retention, moisture sensitive	Blend component; improves antimicrobial activity and film cohesion	[28]
**Other protein-** **Gelatin**	Excellent film formation, biodegradable, good oxygen and light barrier	Hygroscopic, poor thermal stability, brittle	Blend component; improves flexibility and compatibility with pea protein	[8]
**Polysaccharide-** **Pectin**	GRAS, edible, biodegradable, gel-forming	Weak mechanical properties, poor water resistance	Blend component; improves film formation and shelf-life extension	[32]
**Polysaccharide-** **Starch**	Renewable, inexpensive, biodegradable, excellent film-forming ability	Hydrophilic, poor moisture barrier, brittle	Co-matrix (often the major phase, e.g., 80–95% starch: 5–20% PPI in extruded films); enhances processability but usually needs modification	[33]
**Synthetic/biodegradable—** **PLA**	High mechanical strength, transparency, compostability	Moderate moisture and gas barrier, brittleness	Typically a structural/laminate layer rather than a true blend with pea protein; improves strength and dimensional stability	[18]
**Synthetic/biodegradable—** **PHA**	Hydrophobicity, biodegradablity, good gas barrier	Production cost remains higher than conventional commodity plastics in many current production scenarios; however, this limitation is scale- and process-dependent and varies according to feedstock, fermentation efficiency, and purification requirements	Mainly used as a structural or coating layer rather than a direct blend component; improves moisture resistance, durability, and mechanical stability of protein-based films	[1]
**Synthetic/biodegradable—** **PBS**	Good processability, compostability, moisture resistance	Moderate gas barrier	Structural/blend layer; enhances flexibility and processing performance	[2]

**Table 4:** Comparison of conventional edible film and coating application methods.

Method	Principle	Advantages	Limitations	Food Application
Wrapping	Preformed film is prepared separately and wrapped around the food [14].	Uniform film thickness, easy characterisation, suitable for packaging.	Requires sealing; limited adhesion to irregular surfaces.	Fruits [3], vegetables, meat [8], cheese [17].
Immersion (Dipping)	Food is immersed in coating solution for a predetermined time and dried [32].	Simple, inexpensive, complete surface coverage.	Thick coating layer; may reduce gas exchange.	Fresh fruits and vegetables [3].
Spray coating	Coating solution is atomised through a nozzle and deposited as fine droplets.	Uniform thin coating, rapid application, suitable for automation [7].	Requires viscosity optimisation and specialised equipment.	Fruits, vegetables, bakery products [7].
Fluidised bed coating	Food particles are suspended by upward airflow while coating is sprayed.	Excellent coating uniformity efficient drying, continuous processing [31].	Suitable mainly for particulate products; high equipment cost.	Nuts, seeds, cereals [31], confectionery.
Pan coating	Products rotate in a coating pan while coating is sprayed and dried with hot air.	High productivity, uniform coating, easy scale-up [3].	Limited to spherical or oval products.	Candies, nuts [31], dried fruits.

**Table 5:** Normalised comparison of additive-loading effects on tensile strength and water-vapour permeability (WVP) across the pea-protein-film studies reviewed in this article, with proposed structure–property mechanisms.

Base System/Additive	Additive Loading Tested	Tensile Strength (MPa)	WVP (Relative to Control)	Proposed Mechanism	References
PPI + tannic acid	0 → 10% (*w*/*w*)	3.86 → 6.45 (increase)	−87.9% at 10%	Phenolic-protein hydrogen bonding and cross-linking densify the network, restricting both chain mobility and water diffusion pathways simultaneously	[43]
Pea protein/corn starch + green tea polyphenols	0 → 8% → 12%	Increases to 46.07 at 8%, declines above 8%	Minimum at 8% (8.25 × 10^−8^), rises above 8% (12.24 × 10^−8^ at 12%, exceeding control)	Hydrogen bonding increases cross-link density up to an optimum; excess polyphenol introduces additional free hydroxyl groups that re-increase hydrophilicity and disrupt the network	[33]
Pea protein/chitosan + Aronia melanocarpa polyphenols	0 → 4% → 6%	Maximum 5.15 +/− 0.22 at 4%, declines above 6%	Minimum at 4%, increases above 4%	Same optimum-loading behaviour as green tea polyphenols above; consistent with a critical phenolic-hydroxyl-to-protein ratio beyond which excess phenolics act as a hydrophilic plasticiser rather than a cross-linker	[44]
Pea starch/soy protein isolate + eugenol-zein nanoparticles	0 → 3%	~1.7-fold increase at 3%	Reduced from 4.59 × 10^−10^ to 3.04 × 10^−10^ g/(s.m.Pa)	Nanoparticles act as a rigid reinforcing phase and create a more tortuous diffusion path (nanocomposite reinforcement mechanism), rather than as hydrogen-bond donors	[28]
PPI + garlic essential oil	up to 2%	No significant change	No significant effect	Garlic-oil organosulfur compounds (e.g., allicin/diallyl disulfide) are volatile and only weakly polar; they partition into the hydrophobic microdomains of the protein matrix without disrupting the hydrogen-bonded backbone	[41]
PPI + cinnamon essential oil	up to 2%	Marked decrease	Marked decrease in permeability (increased hydrophobicity)	Cinnamaldehyde is a polar, reactive aldehyde that can hydrogen bond with and sterically interpose between protein chains, plasticising the network (lowering TS) while its hydrophobic aromatic ring lowers overall film polarity (lowering WVP)	[41]
PPI + thyme essential oil (nano- vs. microemulsion)	0 → 2%	Slight decrease; EB minimum 39.44% at 2%	Increases above 1.0%, most at 2.0%; nanoemulsion outperforms microemulsion	Smaller nanoemulsion droplets disperse more homogeneously and disrupt the protein network less than coarser microemulsion droplets, which create localised discontinuities and moisture pathways	[42]

## Data Availability

The original contributions presented in this study are included in the article. Further inquiries can be directed to the corresponding author(s).
